# Identification of cardiac progenitors that survive in the ischemic human heart after ventricular myocyte death

**DOI:** 10.1038/srep41318

**Published:** 2017-01-25

**Authors:** Mariko Omatsu-Kanbe, Nozomi Nozuchi, Yuka Nishino, Ken-ichi Mukaisho, Hiroyuki Sugihara, Hiroshi Matsuura

**Affiliations:** 1 Department of Physiology, Shiga University of Medical Science, Otsu, Shiga, 520-2192, Japan; 2 Department of Pathology, Shiga University of Medical Science, Otsu, Shiga, 520-2192, Japan

## Abstract

Atypically-shaped cardiomyocytes (ACMs) are beating heart cells identified in the cultures of cardiomyocyte-removed fractions obtained from adult mouse hearts. Since ACMs spontaneously develop into beating cells in the absence of hormones or chemicals, these cells are likely to be a type of cardiac progenitors rather than stem cells. “Native ACMs” are found as small interstitial cells among ventricular myocytes that co-express cellular prion protein (PrP) and cardiac troponin T (cTnT) in mouse and human heart tissues. However, the endogenous behavior of human ACMs is unclear. In the present study, we demonstrate that PrP^+^ cTnT^+^ cells are present in the human heart tissue with myocardial infarction (MI). These cells were mainly found in the border of necrotic cardiomyocytes caused by infarcts and also in the hibernating myocardium subjected to the chronic ischemia. The ratio of PrP^+^ cTnT^+^ cells to the total cells observed in the normal heart tissue section of mouse and human was estimated to range from 0.3–0.8%. Notably, living human PrP^+^ cTnT^+^ cells were identified in the cultures obtained at pathological autopsy despite exposure to lethal ischemic conditions for hours after death. These findings suggest that ACMs could survive in the ischemic human heart and develop into a sub-population of cardiac myocytes.

Adult mammalian heart comprises several cardiac stem or progenitor cells, although cardiomyocytes do not actually multiply to substitute new cells for damaged ones. Therefore, to develop a new method for repairing the injured heart, a number of studies have been reported to manipulate these cells to differentiate into functional cardiomyocytes^1^,^2^,^3^,^4^,^5^,^6^,^7^. Pluripotent stem cells, including bone marrow-derived mesenchymal stem cells (MSCs) and induced pluripotent stem cells (iPSCs) are also important sources for the cardiac regeneration^8^,^9^,^10^,^11^. Usually, such stem cells can be identified based on the expression of stem cell marker protein(s) and/or the characteristics of the stemness, thus allowing for the growth of these cells to differentiate into cardiomyocytes *in vitro* and *in vivo* transplantation.

Atypically-shaped cardiomyocytes (ACMs) are a type of cardiac progenitors identified in the cultures of cardiomyocyte-removed fraction obtained from mouse cardiac ventricles that spontaneously develop into beating cells within 3–5 days culture^12^,^13^,^14^. Beating ACMs possess a peculiar morphology far different from a typical cardiomyocytes and the electrophysiological properties similar to those of sino-atrial (SA) nodal pace maker cells^12^. ACMs can be obtained from neonatal to aged heart, while preserving the expression of the fetal cardiac gene products, such as atrial natriuretic peptide (ANP) and T-type Ca^2+^ channel Ca_V_3.2^13^, and are more ischemic resistant compared with the ventricular myocytes^14^. ACMs are thus thought to be not classified into stem cells but some kinds of cardiac progenitors that already entered into a cardiomyocyte lineage. Recently, cellular prion protein (PrP), also known as CD230, has been reported to serve as a surface marker for isolating cardiac progenitors in differentiating embryonic stem cells (ESCs)^15^. We found that *native ACMs* marked by the co-expression of PrP and contractile protein, such as cardiac troponin T (cTnT), are localized in the interstitial spaces among ventricular myocytes in mouse and also in human heart^16^. However, the physiological role of ACMs has yet to be elucidated.

The aim of the present study was to examine the morphological analysis whether native ACMs could survive in the ischemic human heart. The results show that PrP^+^ cTnT^+^ cells are present in the infarcted area of MI and in the hibernating area subjected to chronic ischemia of human heart tissue specimens. We also demonstrate that the living human PrP^+^ cTnT^+^ cells, assumed to be ACMs, are identified in the cultures prepared from heart tissue dissected from the patients for the pathological autopsy approximately 2 h after death. The findings indicate that some of the ACMs in human heart could survive even after the adjacent ventricular myocytes die under pathophysiological conditions.

## Results

### Beating ACMs prepared from mouse heart

**Figure 1:**
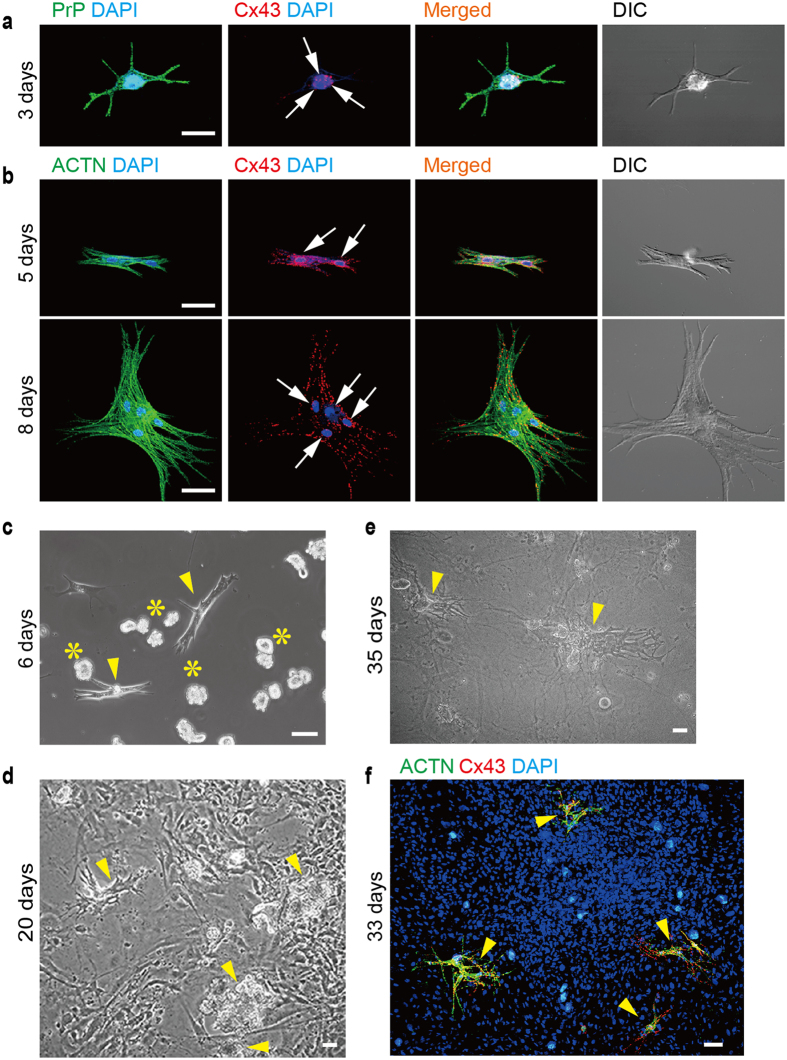
Beating ACMs in the cultures obtained from mouse heart. (**a**) Confocal laser scanning microscopy of double-immunostaining for PrP (green) and Cx43 (red), DAPI staining for nuclei (blue) and DIC images in ACMs obtained from mouse heart cultured for 3 days. Arrows indicate nuclei. Bar, 25 μm. (**b**) Double-immunostaining for ACTN (green) and Cx43 (red) in ACMs cultured for 5 (upper panels) and 8 (lower panels) days. Bar, 50 μm. (**c–e**) Phase contrast image of beating ACMs (arrowheads) co-cultured with ventricular myocyte fraction obtained from the same heart, cultured for 6 (**c**), 20 (**d**) and 35 (**e**) days. Asterisks indicate shortened ventricular myocytes with popcorn-like structures. See movies in Supplementary Movie 1 for (**d**) and Supplementary Movie 2 for (**e**). Bar, 50 μm. (**f**) Merged image of immunostaining for ACTN (green) and Cx43 (red) and DAPI staining in ACMs co-cultured with the ventricular myocyte fraction cultured for 33 days. ACMs indicated by arrowheads were beating before fixation. Bar, 50 μm.

Mouse ACMs started to beat, grow in size and change the shape after 3 days’ culture (Fig. 1a) and usually possess multiple nuclei mostly due to the cell fusion^16^. Connexin 43 (Cx43) is a major gap junction protein in the cardiac ventricles^17^, but not in the SA node^18^, localizing at the intercalated discs of ventricular myocytes. Immunofluorescent signals of cellular prion protein (PrP) were strongly detected at the plasma membrane, while those of Cx43 were detected in the peri-nuclear area of PrP^+^ cells (Fig. 1a), indicating that the cells at this stage express Cx43 particularly near the nucleus. Cx43 expression in beating ACMs gradually increased and spread towards the cell periphery from 5 to 8 days of culture along with the morphological maturation confirmed by the expression of contractile protein α-actinin (ACTN) (Fig. 1b). These observations are similar to the reports that the distribution of Cx43 in cardiomyocytes are changed during the culture^19^ and also compensated and decompensated cardiac hypertrophy^20^.

To determine whether the ACMs could form the gap junction structure with the neighboring cells, the cells were co-cultured with ventricular myocytes obtained from the same heart. However, after a 5 days’ culture, almost all ventricular myocytes had shortened to form popcorn-like structures without adhering to the culture dish, while ACMs tightly adhered to the bottom of the culture dish and spontaneously beat (Fig. 1c). No viable ventricular myocytes were detected, even in independent cultures, over these 6 days of culture^14^. Instead, fibroblast-like cells originating from the endogenous heart tissue, such as cardiac fibroblasts or dedifferentiated cardiomyocytes^21^,^22^, proliferated, becoming nearly confluent after culture for 20 days (Fig. 1d). ACMs scattered across the same culture dish continued to beat, and their individual pulses rhythmically pulling the fibroblast-like cell sheet, as shown in the movie (Supplementary Movie S1). Large cell assemblies consisting of fused ACMs (Fig. 1e) also beat in synchronous rate independent of the adjacent fibroblast-like cells, as shown in the movie (Supplementary Movie S2). The evidence that ACTN and Cx43 were detected only in ACMs among the confluent cells (Fig. 1f) further supports that the spontaneous beating of ACMs was independent of adjacent fibroblast-like cells.

### Gene expression analyses of ACMs compared with ventricular myocytes prepared from mouse heart

To examine the characteristics of ACMs, the gene expression profiles of ACMs were compared with those of ventricular myocytes. Cardiac myocyte-removed fraction cells obtained form mouse heart were cultured on plastic films, after which ACMs were selectively collected using laser microdissection and the RNA was extracted. As only ~1,000 ACMs were harvested from the three hearts, the RNA was amplified prior to the genome-wide gene expression analyses using a SurePrint G3 Mouse GE 8 × 60 K microarray (Agilent). The RNA of isolated ventricular myocytes extracted from the same hearts from which the ACMs were prepared was used as a control.

A functional analysis, which was generated using DAVID Bioinformatic Resources (v6.7)^23^, of 358 upregulated genes with a fold change of ≥4 (log_2_ ≥ 2) resulted in 123 clusters, and only three statistically significant categories with *p* < 0.05 and FDR < 0.05 were obtained, termed acetylation, M-phase and ribosome (genes belonging to each category are listed in Supplementary Table S1). In contrast, the functional analysis of 734 downregulated genes with a fold change of ≤4 (log_2_ ≤ 2) resulted in 188 clusters, with statistically significant categories in the top three clusters (Clusters 1–3) exhibiting an enrichment score of >6.0 (Supplementary Table S2). The gene lists of these clusters (Supplementary Table S3) indicate that the expression of genes related to mitochondrial functions and contractile proteins is significantly reduced in ACMs. Transmission electron microscopic (TEM) analyses demonstrated that the amount of mitochondria and myofibers in ACMs are much less than that observed in ventricular myocytes^14^, consistent with the microarray data. One reason for the paucity of mitochondria in ACMs may be the effect of constitutively active autophagy, which results in the sequential digestion of organelles within autophagosomes fused with lysosomes observed in the TEM images^14^. The data thus suggest that ACMs are fundamentally similar to ventricular myocytes though the expression of RNA related to mitosis and ribosome are higher than those of ordinary cardiomyocytes.

### Effect of simulated lethal ischemia on the development of mouse ACMs

We previously demonstrated that ~50% of ACMs which underwent simulated lethal ischemia (precipitated cells were covered by mineral oil for 90 min) could develop into beating cells during the subsequent culture under normoxia while only ~15% of ventricular myocytes could survive the same ischemic condition^14^, thus suggesting that ACMs are more resistant to severe ischemia than ventricular myocytes. The primary factor mediating a series of adaptive responses to low oxygen tension is considered to be an oxygen-sensitive transcriptional activator hypoxia-inducible factor-1 (HIF-1), specifically the oxygen-regulated α subunit of HIF-1 (HIF-1α)^24^. The HIF-1α protein has a short half-life, typically resulting in no detectable protein in normal cells, and is highly regulated by oxygen depletion^25^. Immunofluorescent signals for HIF-1α were clearly detected in most of the normoxic and ischemic-surviving ACMs (Supplementary Fig. S1a), thus displaying immunofluorescent signals that were either similar or somewhat higher in the post-ischemic preparations. As expected, the expression of HIF-1α was negligible in isolated ventricular myocytes under normoxia conditions, and such expression was also weak in the post-ischemic preparations (Supplementary Fig. S1b).

### Presence of native ACMs in the human heart with MI

**Figure 2:**
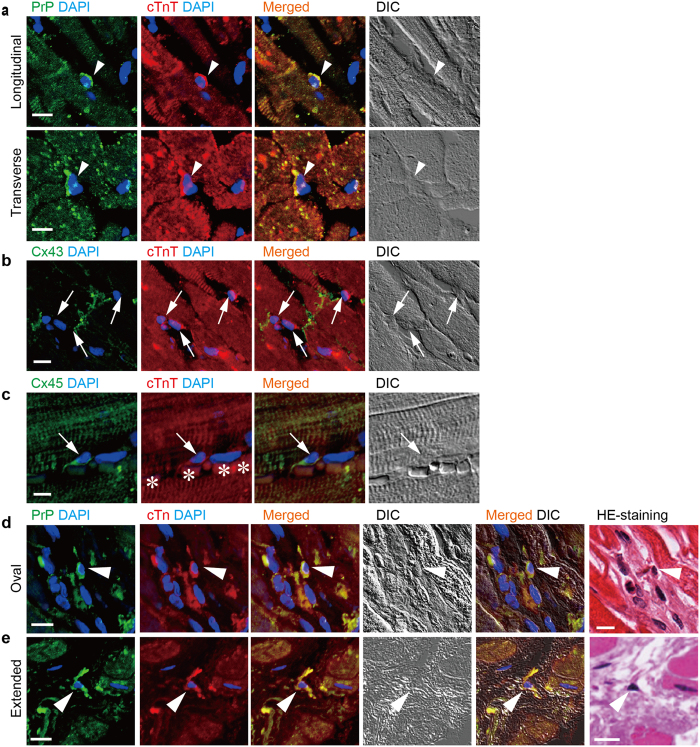
Presence of PrP^+^ cTnT^+^ cells in the normal and the infarcted human heart. (**a**) Double-immunostaining for PrP (green) and cTnT (red), DAPI staining (blue) and DIC images in longitudinal (upper panels) and transverse (lower panels) sections of normal human heart tissue. Arrowheads indicate PrP^+^ cTnT^+^ cells. Bar, 10 μm. (**b**) Double-immunostaining for Cx43 (green) and cTnT (red) in normal human heart tissue. Arrows indicate Cx43^-^cTnT^+^ cells. Bar, 10 μm. (**c**) Double-immunostaining for Cx45 (green) and cTnT (red) in normal human heart tissue. Arrow indicates Cx45^+^ cTnT^+^ cell. Asterisks show erythrocytes. Bar, 10 μm. (**d**,**e**) Double-immunostaining for PrP (green) and cTnT (red) in human myocardium with infarction. Immunostained heart tissue sections were subsequently stained with HE reagent to obtain immunofluorescent and HE staining images from the same tissue section. Arrowheads indicate PrP^+^ cTnT^+^ cells with oval (**d**) and extended (**e**) shapes. HE staining images with lower magnifications are shown in Fig. 3. Bar, 10 μm.

In the normal human heart tissue specimens, native ACMs (PrP^+^ cTnT^+^ cells) were localized in the interstitial spaces or packed into the small spaces among ventricular myocytes as one of the small cells, found in both longitudinal and transverse tissue sections (Fig. 2a). Immunostaining analysis revealed that at least cTnT^+^ cells resident in the interstitial spaces among the ventricular myocytes were not detected to express Cx43 (Fig. 2b), similar to the observations in mouse preparations^16^. In contrast, some of the interstitial cTnT^+^ cells were found to express Cx45 (Fig. 2c), a major gap junction protein isoform in the primary and secondary pacemaker area of the SA node^18^, thus indicating that native human ACMs are more similar to pacemaker cells than to ventricular myocytes.

**Figure 3:**
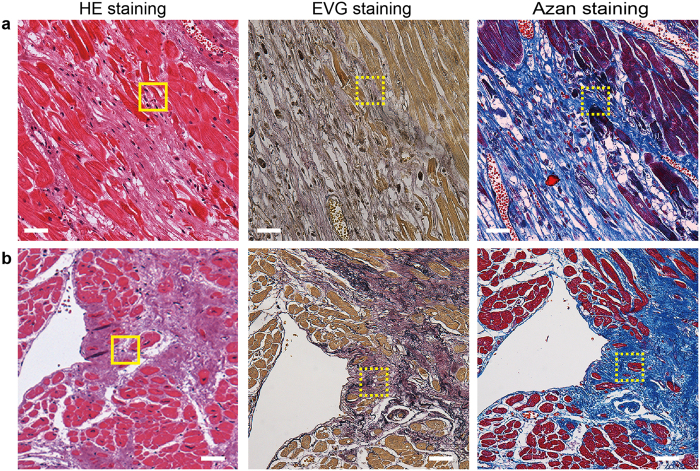
Staining of collagenous tissue in the infarcted human heart. Left panels indicate the lower magnification of HE staining shown in Fig. 2. Solid squares in (**a** and **b**) indicate the area enlarged in Fig. 2d and e, respectively. EVG (middle panels) and Azan (right panels) staining images obtained from one of the sections of a series of tissue sectioning from the same heart tissue of the HE staining. Dotted squares in EVG and Azan staining images are near area in z-axis direction where PrP^+^ cTnT^+^ cells was observed. Bar, 50 μm.

The evidence that mouse ACMs are ischemic resistant in *in vitro* experiments^14^ suggests that ACMs might be more likely to survive in an ischemic human heart than ventricular myocytes. To determine whether or not native ACMs were able to survive in the human ischemic heart, we examined the presence of PrP^+^ cTnT^+^ cells in infarcts of various sizes and ages in human heart tissue specimens obtained from 5 patients in whom MI was found based on the findings of a pathological autopsy (Table 1). PrP^+^ cTnT^+^ cells were usually found in the border of necrotic cells of swollen eosinophilia caused by MI with oval shapes (Fig. 2d); however, in some cases, the cells had extended their membrane toward the space where the microenvironment had been destroyed (Fig. 2e). Figure 3a,b demonstrate the EVG and Azan staining images to visualize collagenous scar in the sections close to the area where the oval- and extended-shaped PrP^+^ cTnT^+^ cells were found, respectively. In the present study, PrP^+^ cTnT^+^ cells were detected near the necrotic cells in the infarct area, but they were rarely found in the center of the fibrous tissue or collagenous scar tissue in human heart tissue specimens.

### Number of PrP^+^ cTnT^+^ cells in mouse and human cardiac ventricular tissues

**Figure 4:**
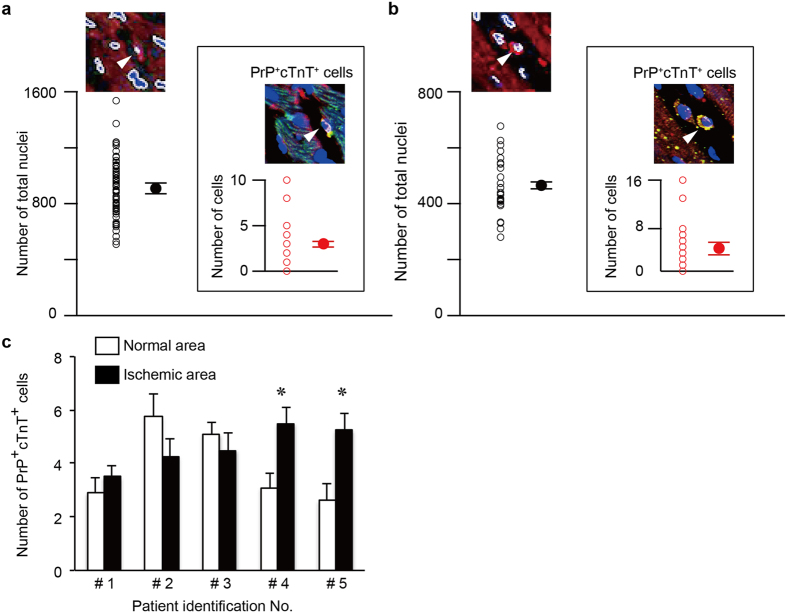
Number of PrP^+^ cTnT^+^ cells in mouse and human cardiac ventricular tissues. (**a**,**b**) Cell number was counted in the image field (1,024 × 1,024 pixels; 636.5 × 636.5 μm with a 20× objective) of double-immunostaining for PrP and cTnT in normal mouse (**a**) and normal human heart (**b**) tissue sections. The number of nuclei was assumed to be the total number of cells. The nuclei marked by white lines in the merged images of cTnT and DAPI (upper panels) were counted. Inserts indicate PrP^+^ cTnT^+^ cells counted in the merged images of PrP, cTnT, and DAPI (upper panel in the insert). Arrowhead indicates PrP^+^ cTnT^+^ cells. Open circles indicate individual values, and closed circles indicate the mean ± S.E.M. Data were obtained from 72 fields from 3 mice (**a**) and 28 fields from 3 hearts without pathological changes (Supplementary Table S4) (**b**). (**c**) Numbers of PrP^+^ cTnT^+^ cells counted in normal and ischemic areas from the same human heart tissue specimens obtained from five patients diagnosed with MI by pathological autopsy. Normal areas were selected where distanced from the ischemic area. Patient identification numbers #1–#5 refer to a male aged 86 years, a female aged 85 years, a female aged 82 years, a female aged 41 years, and a female aged 51 years, respectively (Table 1). Each value represents the mean ± S.E.M. from 10 fields. *p < 0.05 vs. normal area.

To estimate the ratio of PrP^+^ cTnT^+^ cells to total cells in heart tissue, the number of PrP^+^ cTnT^+^ cells was counted in an image field acquired by confocal laser scanning microscopy with double-immunostaining for PrP and cTnT. The number of nuclei, assumed to be the total number of cells, was counted using computer software (Fig. 4a,b). In mouse cardiac ventricular tissue, the numbers of total and PrP^+^ cTnT^+^ cells were 911 ± 23.9 and 3 ± 0.2 cells, respectively (mean ± S.E.M. of 72 fields from 3 mice, Fig. 4a), while numbers in normal human cardiac ventricular tissue without pathological changes (the patient data are shown in Supplementary Table S4) were 466 ± 18.7 and 4.8 ± 0.7 cells, respectively (mean ± S.E.M. of 28 fields from 3 patients, Fig. 4b). The ratio of PrP^+^ cTnT^+^ cells to the total cells was thus estimated to range from 0.3–0.8% in mouse and normal human heart tissue specimens.

We next compared the number of PrP^+^ cTnT^+^ cells between the normal and the infarcted areas of the same human ventricular tissues. The samples were selected from human heart tissue specimens with a relatively small size of infarcts found at autopsy but not those with a wide range of infarcts. The clinicopathological data of 5 patients (patient #1–#5), the basic information of the patients, coronary lesions, infarct areas, associated diseases, cause of death, clinical intervention for coronary lesions and medication, are listed in Table 1. The normal areas in the same heart were selected from sites located at least ~8–10 mm from the edge of the infarcted area where the ventricular myocytes appeared to be morphologically normal. The number of PrP^+^ cTnT^+^ cells between the normal and infarcted areas of the same human heart tissue specimens obtained from 5 patients (Table 1), counting approximately 2–6 and 3–6 cells in the normal and infarcted areas of the myocardium, respectively (Fig. 4c). While two of the five heart tissue specimens (patients #4 and #5) showed a statistically significant increase in the number of PrP^+^ cTnT^+^ cells in the infarcted area compared with the normal tissue, no significant changes were seen in the other three specimens (patients #1~#3), suggesting the individual differences between the patients. Therefore, it is difficult to conclude that the ischemic stress induces the proliferation of the native ACMs in the human heart.

### PrP^+^ cTnT^+^ cells in the hibernating myocardium

**Figure 5:**
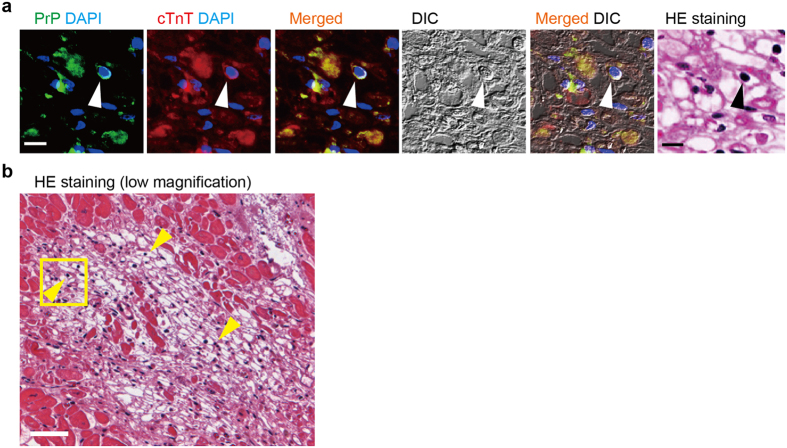
Presence of PrP^+^ cTnT^+^ cells in the hibernating human myocardium. (**a**) Double-immunostaining for PrP (green), cTnT (red), DAPI staining (blue), and DIC and HE staining images in the hibernating human myocardium subjected to chronic ischemia. Immunostained heart tissue sections were subsequently stained with HE reagent to obtain immunofluorescent and HE staining images from the same tissue section. Arrowhead indicates PrP^+^ cTnT^+^ cell. Bar, 10 μm. (**b**) Lower magnification of HE staining. Square indicates the area enlarged in (**a**). Arrowheads indicate representative PrP^+^ cTnT^+^ cells confirmed by immunostaining. Bar, 50 μm.

There is a peculiar region, defined as the hibernating myocardiun the human heart displaying vacuolated or empty cells, but the nuclei remain intact, consisting of non-contractile myocytes to adapt to chronic ischemia^26^,^27^,^28^,^29^,^30^. Unlike infarcted myocardium, the cells in the hibernating myocardium remain viable even after losing myofibers and recover their functionality when reperfusion occurs. The absence of any neutrophil infiltration also proved the viability of the cells in this area. We found the presence of PrP^+^ cTnT^+^ cells in the hibernating myocardium that distributed from the center to the edge of such specific area (Fig. 5). Ventricular myocytes in the hibernating area are degenerated without organized contractile proteins, due to the loss of myofibers (Fig. 5a). The adjacent myocardium appeared coagulative necrosis with eosinophilia caused by MI (Fig. 5b). The observations suggest that the native ACMs can survive under conditions of chronic mild ischemia, where cardiomyocytes are forced into hibernation.

### Living PrP^+^ cTnT^+^ cells prepared from the normal human heart

**Figure 6:**
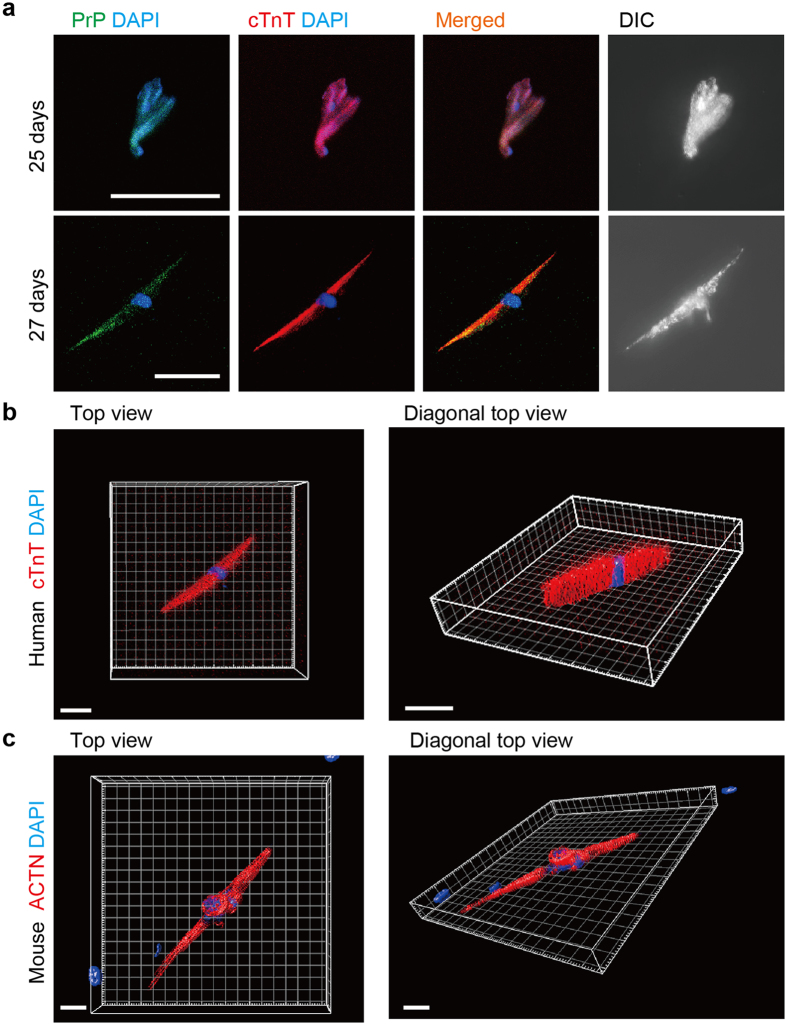
Human PrP^+^ cTnT^+^ cells identified in the cultures. (**a**) Double-immunostaining for PrP (green), cTnT (red), DAPI staining (blue), and DIC images in PrP^+^ cTnT^+^ cells obtained from human heart tissue without pathological changes by autopsy cultured for 25 (upper panels) and 27 (lower panels) days. Bar, 50 μm. (**b**) 3D views with surface rendering images of z-stacked confocal laser scanning microscopy of immunofluorescent staining for cTnT (red) and DAPI staining (blue) in human PrP^+^ cTnT^+^ cells cultured for 27 days shown in (**a**). Top (bar, 30 μm) and diagonal top (bar, 40 μm) views are displayed. (**c**) 3D views with surface rendering images of immunofluorescent staining for ACTN (red) and DAPI staining (blue) in mouse ACMs cultured for 7 days. Top (bar, 20 μm) and diagonal top (bar, 20 μm) views. The grid width in (**b**) and (**c**) is 10 μm each.

We next examined whether living human ACMs could be identified in the culture. The cardiomyocyte-removed fractions were prepared from human heart tissue, without diagnosis of heart diseases, provided by the pathological autopsy within 2 h after the patients died and cultured in the semisolid-culture medium. Unlike the cell fractions prepared from freshly dissected mouse cardiac tissues, a very small number of cells was detected in the cultures obtained from human myocardium hours after death. Finally, we identified few cells with peculiar morphology expressing PrP and cTnT after ~25 days’ culture that survived the severe ischemic period after death (Fig. 6a). Although these cells were not observed to beat, the expression of cardiac-specific contractile protein cTnT in entire cell image indicates these cells to be human ACMs or ACM-like cells (PrP^+^ cTnT^+^ cells). Three-dimensional (3D) views of the human ACMs or ACM-like cells immunofluorescently labeled for cTnT displayed slim, but thick cells with a single nucleus (Fig. 6b). The similar cell shapes were also found in mouse ACMs cultured for ~7 days (Fig. 6c).

## Discussion

The present study demonstrates that native ACMs survive in the ischemic human heart where the ventricular myocytes died. We also show the presence of expected human ACMs in the culture.

Although the adult mammalian heart is one of the least regenerative organs, pre-existing cardiomyocytes have been shown to be capable of re-entering the cell cycle^31^,^32^,^33^,^34^,^35^,^36^,^37^. However, the degree of cardiomyocyte renewal is far too small-scale to wholly replace dead cells. A number of studies have therefore attempted to overcome this gap and encourage organ regeneration using endogenous cells, such as cardiac stem or progenitor cells and bone marrow-derived MSCs, as well as exogenous cells, such as ESCs and iPSCs (for review^5^,^6^,^38^). Cardiac stem or progenitor cells can be identified by stem cell marker proteins and proliferated and differentiated into cardiomyocytes by treatment with chemicals, such as 5′-azacytidine^2^,^3^, oxytocin^3^,^39^, and trichostatin A^39^, or by transplantation *in vivo*^2^,^10^,^35^,^39^,^40^,^41^. However, the endogenous behavior and physiological role or importance of these cells remains unknown. ACMs are likely distinct cell populations from these previous reported cells, given their lack of stem cell markers and their ability to spontaneously develop into beating cells in the absence of stimuli and lack of any appreciable proliferation in culture^12^,^13^. However, several characteristics of ACMs, such as their spontaneous beating and expression of cardiac-specific proteins, suggest that they are “progeny” of cardiomyocytes.

Bone marrow-derived MSCs have been shown to act as cardiac stem or progenitor cells that can transdifferentiate into cardiomyocytes by treatment with 5′-azacytidine *in vitro*^42^ or injection into the heart^6^,^38^,^43^,^44^. We prepared total bone marrow cells from mouse limb bones and cultured them in the same semi-solid culture medium used for ACMs (Supplementary Fig. S2a–c). Immunostaining analysis revealed that PrP^+^ cTnT^+^ cells were observed after 14 days of plating (Supplementary Fig. S2b), but the cells did not start to grow in size or beat even after being cultured for 36 days (Supplementary Fig. S2c), indicating that ACMs are not likely derived form bone marrow cells.

The observations that HIF-1α proteins are clearly detected in even normoxic ACMs but not in ventricular myocytes (Supplementary Fig. S1) indicate that the degradation mechanisms for HIF-1α are somewhat inhibited or absent in ACMs, which may also support the view that ACMs are more resistant to ischemia than cardiomyocytes. ACMs are present in neonatal to aged hearts while preserving the expressing of fetal cardiac gene products, such as atrial natriuretic peptide (ANP) and voltage-gated T-type Ca^2+^ channel Ca_V_3.2^13^. Since HIF-1α is a master regulator of cellular and developmental O_2_ homeostasis during the fetal stage^45^, the constitutive presence of HIF-1α protein in ACMs may be an additional fetal characteristics of these cells. Recently, hypoxic cycling cardiomyocytes that silently reside in the myocardium and enter cycling, not fusion, in response to hypoxia are found in the adult mouse heart using HIF-1α as fate mapping marker^37^. However, there are several differences between ischemic resistant ACMs and hypoxic cycling cells, that is, ACMs tend to fuse with each other and thus become multinuclear cells^16^ and do not enter cycling even hypoxic conditions^14^, which indicates that these cells are not similar. Since some mitosis-related genes are more highly expressed in ACMs than in ventricular myocytes in mice (Supplementary Table S1), it is not possible to rule out the proliferation ability of ACMs even though the proliferation or cell division of ACMs has not yet been detected. Therefore, at present, ACMs can be classified as a type of cardiac progenitor cell beyond the stage of differentiation and future research should focus on improving the methods to induce ACMs to enter into cell cycling before developing into mature beating cells.

The age of a myocardial infarct can be determined by establishing a timeline of microscopic changes in the infarction^30^,^46^. Ischemic myocardial degeneration is initially reversible while occlusion of the coronary artery is temporal and blood flow recovers within ~30 min; thereafter, progressive loss of viability occurs towards the cell death, resulting in MI. Most human MI occur due to the coalescence of foci of necrosis over a long period of time; as such, human cardiac tissue specimens obtained through pathological autopsy often contain infarcts of various ages. Ischemic myocardial injury then becomes irreversible ~30 min after interruption of coronary blood flow. Nonspecific breakdown of DNA in necrotic cells causes various nuclear changes, such as nuclear shrinkage and increased basophilia and fragmentation of basophilic nuclei, followed by the total disappearing of nuclei over the following one or two days.

Native ACMs could remain for longer periods in the infarcted (Figs 2, 3 and 4) and hibernating (Fig. 5) myocardium as long as the ischemic stress was not fatal for the cells. Under pathophysiological conditions, several compensatory events occur in the human heart to restore cardiac function, such as diverting cardiomyocytes after MI^31^ and increasing the number of stem cell factor receptor (c-kit)-positive cardiac progenitor cells in the failing heart^47^. Therefore, if ACMs do play a role in such compensation in the ischemic heart, these cells might proliferate or migrate toward the damaged area. In the present observations, however, it is unlikely that PrP^+^ cTnT^+^ cells proliferate or accumulate in the ischemic human heart (Fig. 4c). In an adult mouse heart, the number of cardiomyocytes has been estimated to be ~3 × 10^6^ cells^48^ and 56% of the total cells in the heart^49^ and the ratio of the native ACMs to the total cells in the heart was estimated to be ~0.3% (Fig. 4a). However, using a microscope, we usually count ~500 beating ACMs obtained from a mouse cardiac ventricle of 2/3 size in culture, which is thought to be underestimated due to a loss and a damage of the native ACMs during the preparation procedure. The data suggest that only a part of the native ACMs in the mouse heart could develop into “mature” beating cells under the present experimental conditions. There are some limitations associated with our method to evaluate the exact cell number of PrP^+^ cTnT^+^ cells within an infarct area in the present study. We only counted the cell number in the infarcted area two-dimensionally, even though the MI spreads out three-dimensionally in heart tissue. Future study will thus be needed to observe cells in thick tissue slices based on 3D views.

It is notable that living PrP^+^ cTnT^+^ cells were identified in the cell cultures obtained from the normal human heart prepared from the cardiac tissues that underwent lethal ischemic conditions for hours prior to incubation in the appropriate cell preparation solution (Fig. 6). The data indicate that human PrP^+^ cTnT^+^ cells, assumed to be human ACMs, are more resistant against ischemia compared with ventricular myocytes, compatible with those obtained in mouse^14^. Unlike mouse ACMs, we did not find any PrP^+^ cTnT^+^ cells that had already begun to beat. One of the reasons for this is thought to be the difference in the cell preparation methodology between mouse and human cells, that is, the perfusion method for the mouse heart and the chunk method for the human heart. When preparing mouse ACMs, a whole heart was continuously perfused with an enzyme solution via the ascending aorta in a retrograde fashion to gently isolate cardiomyocytes while avoiding any cell damages, thus resulting in a high yield of beating ACMs. In contrast, in human cell preparations, a lump of human heart tissue obtained from the pathological autopsy was minced in pieces with scissors in an enzyme solution, thereby causing much damage to the cells. We could obtain only a small number of mouse ACMs with either weak or no contraction even after long-term culture for ~20 days when using the chunk method, thereby suggesting that the induced mechanical damage had dramatically reduced the viability and function of ACMs. Therefore, improving the method to prepare cells from the human heart tissue specimens should be the focus of future study to obtain more cells and to determine whether such cells possess the ability to spontaneously start beating.

Since the heart is a highly organized organ generating the rhythmic contractions of the cardiomyocytes, native ACMs are believed to reside quiescently in the heart under physiological conditions. Adjacent ventricular myocytes are electrically or chemically coupled with each other mediated through gap junction structures. In mouse cardiac ventricular tissues, the absence of Cx43 expression in interstitial cell (including ACMs)^16^ is likely important to ensure that the impulse conduction system is not disturbed, and the expression of this protein increases during the morphological saturation of ACMs (Fig. 1), similar to that observed in cardiomyocyte recovery *in vitro*^19^ and *in vivo*^20^. In human cardiac ventricular tissues, the absence of Cx43 expression in the interstitial cells (Fig. 2b) is also thought to be important for preventing the formation of gap junction structure between ventricular myocytes and the ACMs under physiological conditions. However, some interstitial cells were detected to express Cx45 in human cardiac ventricles (Fig. 2c), thus indicating the possibility that ACMs could form a gap junction structure between the nodal cells. Future study should focus on the role of Cx45 in ACMs under physiological and/or pathophysiological conditions in human heart. Unexpectedly, human ACMs (PrP^+^ cTnT^+^ cells) in culture did not show branching or projections and possessing bulge(s) (Fig. 6) which are normally found in mouse ACMs (Fig. 1). Mouse ACMs possessing bulges demonstrate multinuclear cells with more complex morphological structures compared with human ACMs. This may be due to the species difference of mouse and human, but an improvement of the preparation methods for the isolation of human ACMs should be studied in future investigations.

ACMs are thus one of the nearest cardiac progenitor cells to mature cardiomyocytes among reported cardiac stem or progenitor cells^5^,^6^,^37^,^38^ resident in the heart throughout its lifespan. Understanding the fate and potential of these cells, including the ischemic resistance, will aid in the development of new approaches to cell therapy and clarification of the pathogenesis of arrhythmia in the injured heart.

## Methods

The present studies have been approved by the institutional ethic committee and have therefore been performed in accordance with the ethical standards laid down in the 1964 Declaration of Helsinki and its later amendments.

### Animal experiments

All experimental protocols were approved by the Institutional Review Board of the Shiga University of Medical Science Animal Care and Use Committee (Approval no. 2015-5-4). The methods were carried out in accordance with the approved guidelines. Adult C57BL/6 J mice were purchased from Charles River Laboratories Japan.

### Human heart tissues

Human heart tissue specimens were obtained from pathological autopsy. All protocols were approved by the Shiga University of Medical Science Ethics Committee (Approval no. 26–95 and 27–35). The methods were carried out in accordance with the approved guidelines. Informed consent for the use of tissue samples for the experiments was obtained from all subjects. Cardiac tissues dissected within 2~3 h after death were selected.

### Solutions and culture medium

Tyrode solution contained the following (in mM): 140 NaCl, 5.4 KCl, 1.8 CaCl_2_, 0.5 MgCl_2_, 0.33 NaH_2_PO_4_, 5.5 glucose, and 5.0 Hepes (pH adjusted to 7.4 with NaOH). The cell isolation buffer contained the following (in mM): 130 NaCl, 5.4 KCl, 0.5 MgCl_2_, 0.33 NaH_2_PO_4_, 22 glucose, 50 μU mL^−1^ bovine insulin, and 25 Hepes (pH adjusted to 7.4 with NaOH). The cell isolation buffer was used to isolate both ventricular myocytes and ACMs. The cell suspension buffer contained Tyrode solution supplemented with 0.2 mg mL^−1^ bovine serum albumin (BSA), 100 μg mL^−1^ penicillin, 0.1 mg mL^−1^ streptomycin, and 15 μg mL^−1^ phenol red. The semisolid culture medium consisted of an 80:20 mixture of methylcellulose-based medium MethoCult^®^ M3231 (STEMCELL Technologies) and Iscove’s modified Dulbecco’s medium (IMDM). The final composition of the cell culture medium was 1% methylcellulose, 30% fetal bovine serum (FBS), 1% BSA, 2 mM L-glutamine, and 0.1 mM 2-mercaptoethanol in IMDM.

### Cell preparation and culture of ACMs from mouse

The method used for the isolation and culture of the cardiomyocyte-removed crude fraction cells has been previously described^12^,^16^. Briefly, mice were killed *via* intraperitoneal injection of an overdose of sodium pentobarbital (>300 mg kg^−1^) with heparin (8,000 U kg^−1^). The heart was quickly excised, placed in ice-chilled Tyrode solution, and then cannulated via the ascending aorta and perfused in a retrograde fashion with Tyrode solution, cell isolation buffer supplemented with 0.4 mM EGTA and then an enzyme solution containing 0.1% collagenase (Class II; Worthington Biochemical), 0.006% trypsin (Sigma-Aldrich), 0.006% protease (Sigma-Aldrich), and 0.3 mM CaCl_2_ in the cell isolation buffer at 37 °C. Both ventricles were cut out at a point 2/3 from the ventricular apex. The atrial tissue-free ventricular tissues were then further digested with the enzyme solution, with the CaCl_2_ level increased to 0.7 mM. The mixture was washed by centrifugation at 14 × *g* (300 rpm) for 3 min and the supernatant fraction was filtered through a 40-μm mesh filter and further centrifuged at 150 × *g* (1,000 rpm) for 5 min. The pelleted cells of the final centrifugation were obtained as cardiomyocyte-removed fraction cells and resuspended in 600 μL cell suspension buffer added to the semisolid culture medium at a 1:10 dilution, mixed with a vortex mixer for 2 s to disperse the cells, plated in high-quality plastic dishes (μ-Dishes; ibidi GmbH), and maintained at 37 °C in a humidified atmosphere of 95% air and 5% CO_2_. Beating ACMs were identified in the culture of the cardiomyocyte-removed fraction cells after ~3 days.

### Preparation and culture of total bone marrow cells

Mice were killed *via* intraperitoneal injection of an overdose of sodium pentobarbital (>300 mg kg^−1^) with heparin (8,000 U kg^−1^). Total bone marrow cells were obtained from the limbs bones and filtered through a 40-μm mesh filter. An aliquot of the bone marrow cells was then suspended in semisolid culture medium and cultured in μ-Dishes.

### Human cell preparation and culture

Human cardiac tissue was provided by the pathological autopsy within ~2 h after death from a patient (56 years old, female) who did not diagnosed with heart diseases from pathological autopsy. A lump of heart tissue of <1 cm cube was excised out from the site ~1 cm from the apex. The tissue was then cut into pieces and digested using an enzyme solution containing 0.1% collagenase (Class II), 0.03% trypsin, 0.03% protease, and 1 mg mL^−1^ BSA in the cell isolation buffer for ~15 min at 37 °C with intermittent pipetting. The incubation mixture was centrifuged at 14 × *g* (300 rpm) for 5 min and the precipitated tissue was further digested with the enzyme solution supplemented with 0.6 mM CaCl_2_ for ~15 min at 37 °C with pipetting. The reaction mixture was centrifuged at 14 × *g* (300 rpm) for 5 min and the precipitate was washed with the isolation buffer supplemented with 1.2 mM CaCl_2_ and 2 mg mL^−1^ BSA. The precipitate fraction was resuspended in the cell suspension buffer, added to the semisolid culture medium at a 1:10 dilution, mixed with a vortex mixer for 2 s to disperse the cells and cultured in μ-Dishes.

The patient was initially diagnosed with diffuse large B-cell lymphoma (DLBCL) of the duodenum and thereafter was administrated eight cycles of chemotherapy and also underwent partial resection of duodenum because the neoplastic cells had formed a duodenal ulcer. However, the patient later developed recurrence in the remnant duodenum again, and gastrointestinal bleeding from the area of recurrence part was poorly controlled, thus requiring repeated blood transfusion. Finally the patient died eight months after the initial diagnosis, because of fatal arrhythmia due to an electrolyte abnormality which occurred in association with renal dysfunction. There were no apparent pathological findings regarding the patient’s heart tissue specimens based on the findings obtained at autopsy.

### Microscopy

Phase-contrast images of ACMs were acquired using an inverted microscope (DIAPHOT 300; Nikon) and a charge-coupled device (RETIGA 2000 R; QImaging). Color images of stained tissue sections were acquired using an Eclipse 90i microscope (Nikon) and a charge-coupled device (MicroPublisher 5.0 RTV; QImaging). Images were then analyzed using Image Pro Plus software (MediaCybernetics Inc.). The sample size of each acquired image was 1,600 × 11,200 pixels (588 × 442 μm with a 20× objective).

### Immunostaining

For the immunocytochemical experiments, cells cultured on μ-Dishes were fixed with 4% paraformaldehyde and permeabilized/blocked with 0.2% Triton X-100 and 10% FBS in PBS. The permeabilized cells were then labeled with the primary antibody and probed with an AlexaFluor^®^ 488- or 568-conjugated secondary antibody (Molecular Probes). The nuclei were stained with 2 μg mL^−1^ 4′,6′-diamino-2-phenylindole (DAPI).

For the immunohistochemical experiments, 3-μm paraffin sections of human heart tissue were deparaffinized with xylene, rehydrated by a series of 100–70% ethanol washes, retrieved with antigen-retrieving solution (1:200 dilution, ImmunoSaver; Nisshin EM Co.) for 45 min at 98 °C and blocked with 0.2% Triton X-100 and 10% FBS in PBS for 2 h. The sections were then incubated with primary antibody overnight at 4 °C and probed with secondary antibody for 2–3 h at room temperature. The nuclei were stained with DAPI, and probed sections were mounted with glass cover slips in a mounting medium (Vectashield; Vector Lab.).

The fluorescent signals were analyzed using the confocal laser scanning system C1si on an Eclipse TE2000-E inverted microscope (Nikon) to acquire images 1,024 × 1,024 pixels (636.5 × 636.5 μm with a 20× objective) in size. The data for mouse tissue reflect representative images obtained from three experiments, and the data for human tissues reflect representative images obtained from three paraffin sections of at least two different parts of the left cardiac ventricle.

### Histological evaluation of MI

The histological evaluation of the MI was performed with hematoxylin and eosin (HE) staining, Elastica van Gieson (EVG) staining and Azan staining. In EVG staining, elastic fibers, nuclei, collagen fibers and other tissue elements are stained in dark purple, black, red and yellow, respectively. In Azan-staining, elastic fibers, nuclei, erythrocytes and other cytoplasm are stained in deep blue, deep red, reddish orange and red, respectively.

### Counting cell numbers

The cell numbers were counted in the 1,024 × 1,024 pixel (636.5 × 636.5 μm with a 20× objective) image field acquired with confocal laser scanning microscopy of heart tissues double-immunostained for PrP and cTnT. The number of PrP^+^ cTnT^+^ cells was counted in the merged images of fluorescently labeled PrP and cTnT. The number of nuclei was assumed to be the total number of cells. The nuclei were marked by white lines in the merged images of cTnT and DAPI and counted using Image Pro Plus software.

### Three-dimensional (3D) analysis of immunofluorescent staining images

Z-stacked images of immunofluorescent staining were acquired using the confocal laser scanning system C1si. The 3D microscopic datasets were then analyzed and visualized with the volume rendering image processing software program Imaris (ver 8.1, Bitplane AG).

### Simulated lethal ischemia assay

A simulated lethal ischemia assay was performed according to the described method^50^ with slight modifications^14^. The cells were centrifuged into a pellet, covered with mineral oil layer and incubated for 90 min at 37 °C. After exposure to the ischemia, the cardiac myocyte-removed fraction cells were resuspended in fresh cell suspension buffer, cultured for 6 days, and the ventricular myocytes were suspended in the cell suspension buffer and incubated for 30 min at 37 °C under normoxia.

### Antibodies

The primary antibodies were mouse monoclonal anti-prion protein (anti-PrP, clone SAF83 for mouse and SAF54 for human samples, 1:100; Bertin Pharma Co.), anti-α-actinin (anti-ACTN, A7811, clone EA-53, 1:400; Sigma-Aldrich) and anti-hypoxia inducible factor-1 α (anti-HIF-1α, MA1–516, clone mgc3, 1:200; Pierce-Thermo), rabbit polyclonal anti-connexin 43 (anti-Cx43, #3512, 1:100; Cell Signaling Technology) and anti-connexin 45 (anti-Cx45, #sc-25716, 1:50; Santa Cruz Biotechnology), and goat polyclonal anti-cardiac troponin T (anti-cTnT, sc-8121, 1:50; Santa Cruz Biotechnology) antibodies.

### Statistical analysis and image data

Data are represented as the mean ± S.E.M. Student’s *t*-test was used for all analyses, and values of *p* < 0.05 were considered to be significant. Image data are representative of three different cell preparations for mice and at least five different acquired images for human heart tissue specimens.

### RNA extraction and microarray

Cardiac myocyte-removed fraction cells obtained from three hearts were collected and cultured on special slides, aluminum frame-slides with polyethylene terephthalate foil (1.4 μm in thickness) (Leica). After 6 days of culture, the cells on the foil were washed with PBS, fixed with 70% ethanol for 15 s at −20 °C and washed with RNase-free water for 15 s. The cells were then stained with 0.05% toluidine blue for 10 s, washed with RNAse-free water for 15 s twice and air-dried. The ACMs, usually 900–1,200 cells in total, on the foil were collected using the LMD7000 laser microdissection system (Leica). RNA was extracted from the collected ACMs using NucleoSpin RNA (Macherey-Nagel). Usually, 0.9–5.0 ng of total RNA of the ACMs was yielded from three hearts. RNA was also extracted from the ventricular myocytes isolated from three hearts that ACMs were prepared, as a control.

The RNAs of both the ACMs and ventricular myocytes were amplified using the Ovation Pico/PicoSL WTA System (NuGEN) to obtain at least 2.0 μg cDNA. Subsequently, genome-wide gene expression analyses were performed using a SurePrint G3 Mouse GE 8 × 60 K Microarray (Agilent). The RNA amplification and expression arrays were performed at Takara Dragon Genomics Center (Mie, Japan).

### Statistical and functional annotation analyses for microarray

The microarray experiments were performed in triplicate from independently prepared RNA collected from three hearts. The statistical analyses and clustering of the functional gene annotation of up- or downregulated genes in the ACMs compared with ventricular myocytes with a log_2_ change of ≥2 (fold change ≥4) were generated using DAVID (database for annotation visualization and integrated discovery) Bioinformatic Resources (v6.7)^23^. Only those terms with a Benjamini-Hochberg corrected Fisher Exact *p*-value of <0.05 and false discovery rate (FDR) of <0.05 were selected for the analyses.

## Additional Information

**How to cite this article**: Omatsu-Kanbe, M. *et al*. Identification of cardiac progenitors that survive in the ischemic human heart after ventricular myocyte death. *Sci. Rep.* **7**, 41318; doi: 10.1038/srep41318 (2017).

**Publisher's note:** Springer Nature remains neutral with regard to jurisdictional claims in published maps and institutional affiliations.

## Supplementary Material

Supplementary Information

Supplementary Movie S1

Supplementary Movie S2

## Figures and Tables

**Table 1:** Clinicopathological data of patients for the heart tissue with MI.

	Patient #1	Patient #2	Patient #3	Patient #4	Patient #5
Age	86	85	82	41	51
Gender	M	F	F	F	F
MI	Subacute and acute	Old	Old	Old	Old
Coronary lesions	Distal LAD (#8) occlusion by plaque rupture, distal RCA (#3 stenosis with plaque rupture)	Proximal LAD (#6)	Distal LAD (#8)	Distal LAD (#10)	Proximal LAD (#6–#7), stenosis of LCX and RCA
Infarct areas	Anteroseptal/inferior area (sampling site) and posterior wall	Anterior wall, subendocardial	Anterolateral area	Anterolateral area	Anteroseptal area
Associated disease (s)	Intractable bronchopneumonia, Prostate cancer and cardiogenic cerebral infarction	Metastatic colony cancer, Diabetes mellitus	Infective endocarditis based on rheumatic heart disease	MDS (post-BM transplantation), Idiopathic interstitial pneumonia	Post-total colectomy hematoma, Diabetic nephropathy
Cause of death	Intractable bronchopneumonia	Metastatic colony cancer	Infective endocarditis	Idiopathic interstitial pneumonia	Post-total colectomy hematoma
Clinical intervention for coronary lesions	No (clinically silent)	Stent insertion in LAD (#6)	No (clinically silent)	No (clinically silent)	Stent insertion in LAD (#6–#7)
Medication	No (clinically silent)	No	No (clinically silent)	No (clinically silent)	No

MI: myocardial infarction. LAD: left anterior descending. RCA: right coronary artery. LCX: left circumflex. MDS: myelodysplastic syndromes. BM: bone marrow.
