# Supplementary material for: Identification of cardiac progenitors that survive in the ischemic human heart after ventricular myocyte death
Source: Sci Rep. 2017 Jan 25;7:41318. doi: 10.1038/srep41318 (PMC5264617; doi:10.1038/srep41318)
Supplement: Supplementary Information [file srep41318-s1.pdf]

Supplementary Information

Identification of cardiac progenitors that survive in the ischemic human heart after ventricular myocyte death

Mariko Omatsu-Kanbe, Nozomi Nozuchi, Yuka Nishino, Ken-ichi Mukaisho,

Hiroyuki Sugihara and Hiroshi Matsuura

Supplementary Table S1. Up-regulated ($log_2 \geq 2$) genes in ACMs compared with ventricular myocytes involved in each functional annotation category with FDR<0.05.

Category 1: SP_PIR_KEYWORDS. Term: acetylation (Count: 75), Benjamini:1.32E-03

Gene Symbol	Gene Name	Genbank
Ddx21	DEAD (Asp-Glu-Ala-Asp) box polypeptide 21	NM_019553
H2afz	H2A histone family, member Z	NM_016750
Smad7	MAD homolog 7 (Drosophila)	NM_001042660
S100a10	S100 calcium binding protein A10 (calpactin)	NM_009112
S100a6	S100 calcium binding protein A6 (calcyclin)	NM_011313
Ugdh	UDP-glucose dehydrogenase	NM_009466
Arpc1b	actin related protein 2/3 complex, subunit 1B	NM_023142
Actn4	actinin alpha 4	NM_021895
Anxa1	annexin A1	NM_010730
Atl3	atlastin GTPase 3	NM_146091
Cdk1	cyclin-dependent kinase 1	NM_007659
Clic1	chloride intracellular channel 1	NM_033444
Cfl1	cofilin 1, non-muscle isoform	NM_007687
Cplx2	complexin 2	NM_009946
Ddah1	dimethylarginine dimethylaminohydrolase 1	NM_026993
Fen1	flap structure specific endonuclease 1	NM_007999
Gmfb	glia maturation factor, beta	NM_022023
G6pdx	glucose-6-phosphate dehydrogenase X-linked	NM_008062
Gsta3	glutathione S-transferase, alpha 3	NM_001077353
Gars	glycyl-tRNA synthetase	NM_180678
Hmox1	heme oxygenase (decycling) 1	NM_010442
S100a4	S100 calcium binding protein A4	NM_011311
Krt18	keratin 18	NM_010664
Lgals3	lectin, galactose binding, soluble 3	NM_001145953
Mthfd2	methylenetetrahydrofolate dehydrogenase (NAD+ dependent)	NM_008638
Mtap1b	microtubule-associated protein 1B	NM_008634
Mcm3	minichromosome maintenance deficient 3 (S. cerevisiae)	NM_008563
Mcm4	minichromosome maintenance deficient 4 homolog (S. cerevisiae)	NM_008565
Msn	moesin	NM_010833
Nphp3	nephronophthisis 3 (adolescent)	NM_028721
Nipsnap3b	nipsnap homolog 3B (C. elegans)	NM_025623
Pgd	phosphogluconate dehydrogenase	NM_001081274
Rpl11	ribosomal protein L11	NM_025919
Glrx3	glutaredoxin 3	NM_023140, BC033506
Mup19	major urinary protein 19	NM_001135127
Rpl7	ribosomal protein L7	NM_011291
Rps4x	ribosomal protein S4, X-linked	BC009100
Hsp90aa1	heat shock protein 90, alpha (cytosolic), class A member 1	NM_010480
Eef1a1	eukaryotic translation elongation factor 1 alpha 1	NM_010106
Mns1	meiosis-specific nuclear structural protein 1	NM_001033865
Rpl6	ribosomal protein L6	NM_011290
Calm3	calmodulin 3	NM_007590
Hmga2	high mobility group AT-hook 2	NM_010441
BC085271	cDNA sequence BC085271	NM_001008551
Pfn1	profilin 1	NM_011072
Prkar2b	protein kinase, cAMP dependent regulatory, type II beta	NM_011158
Rras2	related RAS viral (r-ras) oncogene homolog 2	NM_025846
Rtn4	reticulon 4	NM_194054
Serpinb1a	serine (or cysteine) peptidase inhibitor, clade B, member 1a	NM_025429
Synpr	synaptoporin	NM_173051
Srp19	signal recognition particle 19	NM_025527
Rps25	ribosomal protein S25	NM_024266
Rpl13a	ribosomal protein L13A	NM_009438
Anxa2	annexin A2	NM_007585
Nutf2	nuclear transport factor 2	NM_026532
Cks2	CDC28 protein kinase regulatory subunit 2	NM_025415
Psat1	phosphoserine aminotransferase 1	NM_177420
Ugt1a6b	UDP glucuronosyltransferase 1 family, polypeptide A6B	NM_201410

Supplementary Table S1. (Continued)

Esd	esterase D/formylglutathione hydrolase	BC046766, NM_016903
Spns1	spinster homolog 1 (Drosophila)	NM_023712
Smc2	structural maintenance of chromosomes 2	NM_008017
Txn1	thioredoxin 1	NM_011660
Trip13	thyroid hormone receptor interactor 13	NM_027182
Taldo1	transaldolase 1	NM_011528
Tagln2	transgelin 2	NM_178598
Tmem30a	transmembrane protein 30A	NM_133718
Tpm1	tropomyosin 1, alpha	NM_001164249
Tpm4	tropomyosin 4	NM_001001491
Tubb3	tubulin, beta 3	NM_023279
Ube2c	ubiquitin-conjugating enzyme E2C	NM_026785
Uhrf1	ubiquitin-like, containing PHD and RING finger domains, 1	NM_010931
Uck2	uridine-cytidine kinase 2	NM_030724
Vim	vimentin	NM_011701
Zc3hav1l	zinc finger CCCH-type, antiviral 1-like	NM_172467
Zbtb8os	zinc finger and BTB domain containing 8 opposite strand	NM_025970

Category 2: GOTERM_BO_FAT, Term: M-phase (Count: 19), Benjamini: 2.82E-02

**Gene Symbol**	**Gene Name**	**Genbank**
Dsn1	DSN1, MIND kinetochore complex component, homolog (S. cerevisiae)	NM_025853
Ndc80	NDC80 homolog, kinetochore complex component (S. cerevisiae)	NM_023294
Zwint	ZW10 interactor	NM_025635
Zwilch	Zwilch, kinetochore associated, homolog (Drosophila)	NM_026507
Mki67	antigen identified by monoclonal antibody Ki 67	NM_001081117
Birc5	baculoviral IAP repeat-containing 5	NM_001012273
Cdk1	cyclin-dependent kinase 1	NM_007659
Cdc20	cell division cycle 20 homolog (S. cerevisiae)	NM_023223
Ereg	epiregulin	NM_007950
Mns1	meiosis-specific nuclear structural protein 1	NM_008613
Ncapg2	non-SMC condensin II complex, subunit G2	NM_133762
Hmga2	high mobility group AT-hook 2	NM_010441
Cks2	CDC28 protein kinase regulatory subunit 2	NM_025415
Smc2	structural maintenance of chromosomes 2	NM_008017
Ddx21	DEAD (Asp-Glu-Ala-Asp) box polypeptide 21	NM_027954
Trip13	thyroid hormone receptor interactor 13	NM_027182
Tacc3	transforming, acidic coiled-coil containing protein 3	NM_001040435
Tubb3	tubulin, beta 3	NM_023279
Ube2c	ubiquitin-conjugating enzyme E2C	NM_026785

Caegory 3: KEGG_PATHWAY, Term: Ribosome (Count: 11), Benjamini: 3.86E-03

**Gene Symbol**	**Gene Name**	**Genbank**
Rpl11	ribosomal protein L11	NM_025919
Rpl39	ribosomal protein L39	NM_026055
Rpl7	ribosomal protein L7	NM_011291
Rps4x	ribosomal protein S4, X-linked	BC009100
Rps27a	ribosomal protein S27A	NM_001033865
Rpl6	ribosomal protein L6	NM_011290
Rpl17	ribosomal protein L17	NM_001002239
Rpl36al	ribosomal protein L36A-like	NM_025589
Rps27l	ribosomal protein S27-like	NM_026467
Rps25	ribosomal protein S25	NM_024266
Rpl13a	ribosomal protein L13A	NM_009438

Category: original database where the terms orient.
Term: enriched term associated with increased gene list.
Count: the number of genes involved in each category.
Benjamini: Benjamini-Hochberg corrected Fisher Exact p-value.
FDR: False Discovery Rate.

Supplementary Table S2. Functional annotation clusters of categories including downregulated genes ($log_2 \leq 2$) in ACMs compared with ventricular myocytes with high enrichment scores of >6.0 with FDR<0.05.

Category	Term	Count	Benjamini
**Cluster 1 (Enrichment score: 12.728719312444023)**			
GOTERM_CC_FAT	GO:0005743~mitochondrial inner membrane	49	6.64E-15
GOTERM_CC_FAT	GO:0019866~organelle inner membrane	49	4.00E-14
GOTERM_CC_FAT	GO:0031966~mitochondrial membrane	51	1.40E-12
GOTERM_CC_FAT	GO:0005740~mitochondrial envelope	51	1.32E-11
GOTERM_CC_FAT	GO:0031967~organelle envelope	55	1.21E-08
GOTERM_CC_FAT	GO:0031975~envelope	55	1.29E-08
**Cluster 2 (Enrichment Score: 10.819174127194598)**			
GOTERM_CC_FAT	GO:0043292~contractile fiber	24	8.23E-11
GOTERM_CC_FAT	GO:0030016~myofibril	23	2.32E-10
GOTERM_CC_FAT	GO:0044449~contractile fiber part	22	5.21E-10
GOTERM_CC_FAT	GO:0030017~sarcomere	20	7.51E-09
**Cluster 3 (Enrichment Score: 6.576073300361855)**			
KEGG_PATHWAY	mmu05010:Alzheimer's disease	27	5.42E-06
KEGG_PATHWAY	mmu00190:Oxidative phosphorylation	22	7.12E-06
KEGG_PATHWAY	mmu05012:Parkinson's disease	21	1.94E-05
KEGG_PATHWAY	mu05016:Huntington's disease	24	5.90E-05

Category: original database where the terms orient. Term: enriched term associated with an increased gene list. Count: number of genes involved in each category. FDR: false discovery rate. Benjamini: Benjamini-Hochberg corrected Fisher Exact *p*-value. See Supplementary Table S2 for the lists of genes in each cluster.

Supplementary Table S3. Down-regulated ($log_2 \leq 2$) genes in ACMs compared with ventricular myocytes involved in annotation clusters 1, 2 and 3 with FDR<0.05. (see Fig.1b for categories in each cluster)

Cluster 1 (Count: 55)

Gene Symbol	Gene Name	Genbank
Hmgcl	3-hydroxy-3-methylglutaryl-Coenzyme A lyase	NM_008254
Hmgcs2	3-hydroxy-3-methylglutaryl-Coenzyme A synthase 2	NM_008256
Bdh1	3-hydroxybutyrate dehydrogenase, type 1	NM_175177
Atp5a1	ATP synthase, H+ transporting, mitochondrial F1 complex, alpha subunit	NM_007505
Atp5d	ATP synthase, H+ transporting, mitochondrial F1 complex, delta subunit	NM_025313
Abcb7	ATP-binding cassette, sub-family B (MDR/TAP), member 7	NM_009592
Gchfr	GTP cyclohydrolase I feedback regulator	NM_177157
Hrasls	HRAS-like suppressor	NM_013751
Ndufa1	NADH dehydrogenase (ubiquinone) 1 alpha subcomplex, 1	NM_019443
Ndufa13	NADH dehydrogenase (ubiquinone) 1 alpha subcomplex, 13	NM_023312
Ndufa8	NADH dehydrogenase (ubiquinone) 1 alpha subcomplex, 8	NM_026703
Ndufb7	NADH dehydrogenase (ubiquinone) 1 beta subcomplex, 7	NM_025843
Ndufv1	NADH dehydrogenase (ubiquinone) flavoprotein 1	NM_133666
Ndufv3	NADH dehydrogenase (ubiquinone) flavoprotein 3	NM_030087
Uqcr10	ubiquinol-cytochrome c reductase, complex III subunit X	NM_197979
Acat1	acetyl-Coenzyme A acetyltransferase 1	NM_144784
Acsl1	acyl-CoA synthetase long-chain family member 1	NM_007981
Acadvl	acyl-Coenzyme A dehydrogenase, very long chain	NM_017366
Bckdhb	branched chain ketoacid dehydrogenase E1, beta polypeptide	NM_199195
Crat	carnitine acetyltransferase	NM_007760
Ckmt2	creatine kinase, mitochondrial 2	NM_198415
Cox4i1	cytochrome c oxidase subunit IV isoform 1	NM_009941
Cox7a2l	cytochrome c oxidase subunit VIIa polypeptide 2-like	NM_001159529
Cox6a2	cytochrome c oxidase, subunit VI a, polypeptide 2	NM_009943
Cox8b	cytochrome c oxidase, subunit VIIIb	NM_007751
Cox7a1	cytochrome c oxidase, subunit VIIa 1	NM_009944
Cyc1	cytochrome c-1	NM_025567
Dci	dodecenoyl-Coenzyme A delta isomerase	NM_010023
Mpv17	MpV17 mitochondrial inner membrane protein	NM_008622
Got2	glutamate oxaloacetate transaminase 2, mitochondrial	NM_010325
Gcdh	glutaryl-Coenzyme A dehydrogenase	NM_008097
Gcat	glycine C-acetyltransferase	NM_013847
Hadh	hydroxyacyl-Coenzyme A dehydrogenase	NM_008212
Hadha	hydroxyacyl-Coenzyme A dehydrogenase, alpha subunit	NM_178878
Idh2	isocitrate dehydrogenase 2 (NADP+), mitochondrial	NM_173011
Mpst	mercaptopyruvate sulfurtransferase	NM_138670
Mccc1	methylcrotonoyl-Coenzyme A carboxylase 1 (alpha)	NM_023644
Ogdh	oxoglutarate dehydrogenase (lipoamide)	NM_010956
Polg	polymerase (DNA directed), gamma	NM_017462
Atp5g1	ATP synthase, H+ transporting, mitochondrial F0 complex, subunit c	NM_001161419
Tufm	translation elongation factor, mitochondrial	NM_001163713
Ptgds	prostaglandin D2 synthase (brain)	NM_008963
Rhot2	ras homolog gene family, member T2	NM_145999
Ndufs6	NADH dehydrogenase (ubiquinone) Fe-S protein 6	NM_010888
Cox7c	cytochrome c oxidase subunit VIIc	NM_007749
Slc25a20	solute carrier family 25, member 20	NM_020520
Slc25a34	solute carrier family 25, member 34	NM_001013780
Slc25a42	solute carrier family 25, member 42	NM_001007570
Samm50	sorting and assembly machinery component 50 homolog	NM_178614
Sdhb	succinate dehydrogenase complex, subunit B, iron sulfur (Ip)	NM_023374
Suclg1	succinate-CoA ligase, GDP-forming, alpha subunit	NM_019879
Syne1	synaptic nuclear envelope 1	NM_001079686
Tst	thiosulfate sulfurtransferase, mitochondrial	NM_009437
Timm50	translocase of inner mitochondrial membrane 50 homolog (yeast)	NM_025616
Uqcrc1	ubiquinol-cytochrome c reductase core protein 1	NM_025407

Supplementary Table S3. (Continued)

Cluster 2 (Count: 24)

Gene Symbol	Gene Name	Genbank
Ldb3	LIM domain binding 3	NM_001039076
Abra	actin-binding Rho activating protein	NM_175456
Actn2	actinin alpha 2	NM_033268
Ankrd23	ankyrin repeat domain 23	NM_153502
Itgb1bp2	integrin beta 1 binding protein 2	NM_013712
Jph2	junctophilin 2	NM_021566
Lrrc10	leucine rich repeat containing 10	NM_146242
Pygm	muscle glycogen phosphorylase	NM_011224
Myo18b	myosin XVIIIb	NM_028901
Myh11	myosin, heavy polypeptide 11, smooth muscle	NM_001161775
Myh6	myosin, heavy polypeptide 6, cardiac muscle, alpha	NM_001164171
Myh7	myosin, heavy polypeptide 7, cardiac muscle, beta	NM_080728
Myot	myotilin	NM_001033621
Myoz2	myozenin 2	NM_021503
Nrap	nebulin-related anchoring protein	NM_008733
Obscn	obscurin, cytoskeletal calmodulin and titin-interacting RhoGEF	NM_199152
Pecam1	platelet/endothelial cell adhesion molecule 1	NM_001032378
Slmap	sarcolemma associated protein	NM_032008
Trim63	tripartite motif-containing 63	NM_001039048
Spnb2	spectrin beta 2	NM_175836
Syne1	synaptic nuclear envelope 1	NM_001079686
Synpo2	synaptopodin 2	NM_080451
Tcap	titin-cap	NM_011540
Tnni3	troponin I, cardiac 3	NM_009406

Cluster 3 (Count: 31)

Gene Symbol	Gene Name	Genbank
Atp5a1	ATP synthase, H+ transporting, mitochondrial F1 complex, alpha subunit	NM_007505
Atp5d	ATP synthase, H+ transporting, mitochondrial F1 complex, delta subunit	NM_025313
Atp2a2	ATPase, Ca++ transporting, cardiac muscle, slow twitch 2	NM_009722
Atp6v1b2	ATPase, H+ transporting, lysosomal V1 subunit B2	NM_007509
Ndufa10	NADH dehydrogenase (ubiquinone) 1 alpha subcomplex 10	NM_024197
Ndufa1	NADH dehydrogenase (ubiquinone) 1 alpha subcomplex, 1	NM_019443
Ndufa8	NADH dehydrogenase (ubiquinone) 1 alpha subcomplex, 8	NM_026703
Ndufb7	NADH dehydrogenase (ubiquinone) 1 beta subcomplex, 7	NM_025843
Ndufs8	NADH dehydrogenase (ubiquinone) Fe-S protein 8	NM_144870
Ndufv1	NADH dehydrogenase (ubiquinone) flavoprotein 1	NM_133666
Ndufv3	NADH dehydrogenase (ubiquinone) flavoprotein 3	NM_030087
Uqcr10	ubiquinol-cytochrome c reductase, complex III subunit X	NM_197979
Apoe	apolipoprotein E	NM_009696
Cox4i1	cytochrome c oxidase subunit IV isoform 1	NM_009941
Cox7a2l	cytochrome c oxidase subunit VIIa polypeptide 2-like	NM_001159529
Cox6a2	cytochrome c oxidase, subunit VI a, polypeptide 2	NM_009943
Cox8b	cytochrome c oxidase, subunit VIIIb	NM_007751
Cox7a1	cytochrome c oxidase, subunit VIIa 1	NM_009944
Cyc1	cytochrome c-1	NM_025567
Lpl	lipoprotein lipase; similar to Lipoprotein lipase precursor	NM_008509
Mapt	microtubule-associated protein tau	NM_001038609
Mapk1	mitogen-activated protein kinase 1	NM_011949
Ppargc1a	peroxisome proliferative activated receptor, gamma, coactivator 1 alpha	NM_008904
Ppargc1a	polymerase (RNA) II (DNA directed) polypeptide E	NM_008904
Atp5g1	ATP synthase, H+ transporting, mitochondrial F0 complex, subunit C1	NM_001161419
Gapdh	glyceraldehyde-3-phosphate dehydrogenase	NM_008084
Ndufs6	NADH dehydrogenase (ubiquinone) Fe-S protein 6	NM_010888
Cox7c	cytochrome c oxidase subunit VIIc	NM_007749
Sdhb	succinate dehydrogenase complex, subunit B, iron sulfur (Ip)	NM_023374
Tfam	transcription factor A, mitochondrial	NM_009360
Uqcrc1	ubiquinol-cytochrome c reductase core protein 1	NM_025407

Supplementary Table S4. Clinicopathological data of patients for the normal heart tissues

	**#1**	**#2**	**#3**
Age	78	65	50
Gender	M	M	M
Infarct area	No	No	No
Associated diseases	Gastric cancer, usual interstitial pneumonia	Acute mediastinitis sepsis	Acute pancreatitis sepsis

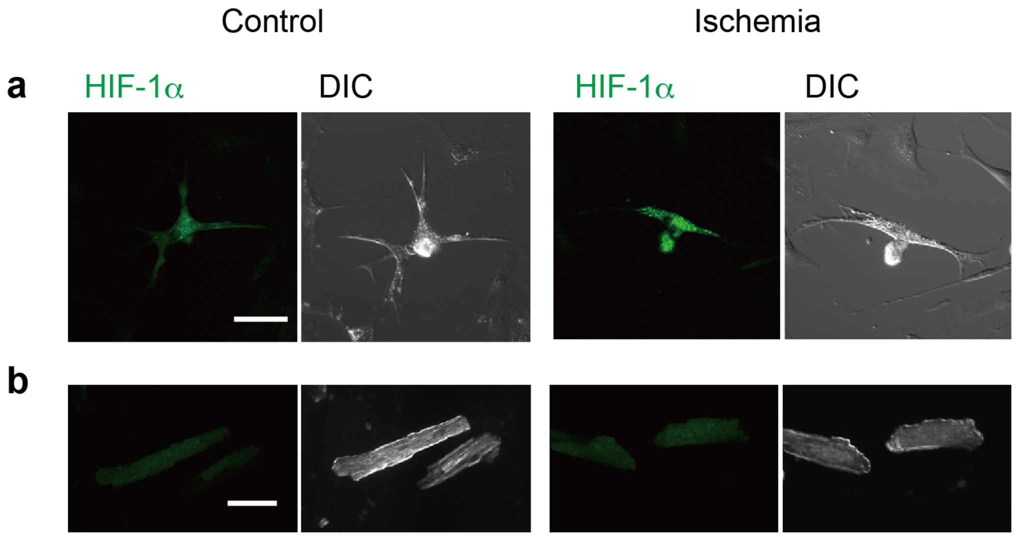

Supplementary Figure S1. Immunostaining for HIF-1α in mouse ACMs and ventricular myocytes. Immunostaining for HIF-1α (green) and DIC images in ACMs (**a**) and isolated ventricular myocytes (**b**) with or without (control) pre-exposure to the simulated lethal ischemia. Bar, 50 μm.

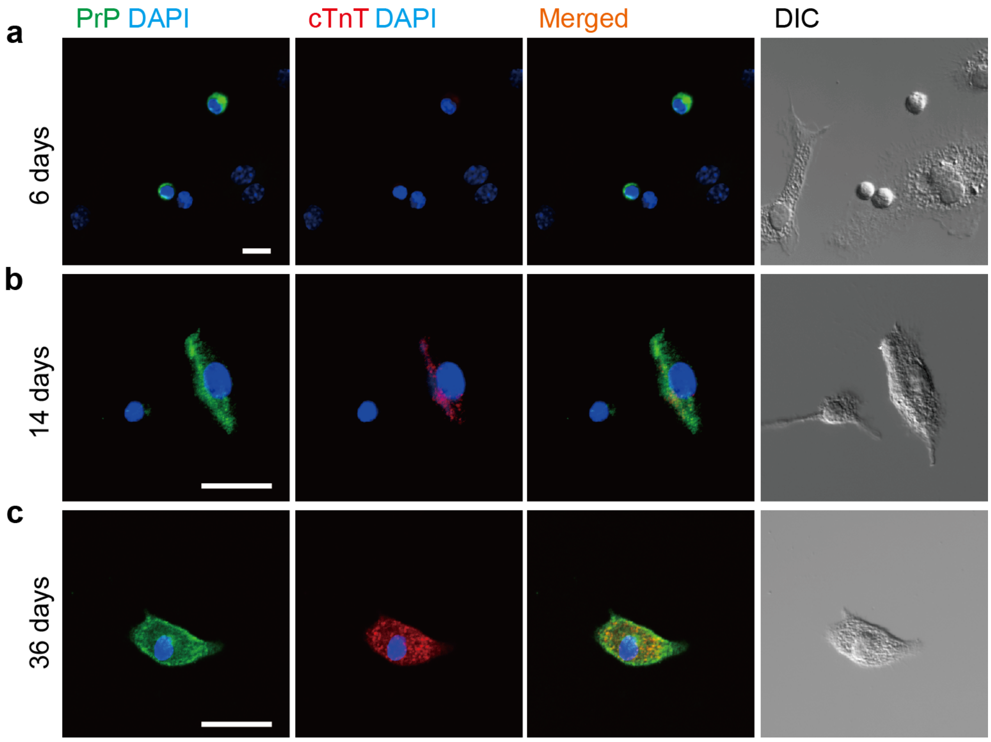

Supplementary Figure S2. Immunostaining for PrP and cTnT in total bone marrow cells in the cultures. Double-immunostaining for PrP (green), cTnT (red), DAPI staining (blue) and DIC images in mouse total bone marrow cells cultured in semi-solid culture medium for 6 (**a**), 14 (**b**) and 36 (**c**) days. **a** Bar, 10 µm. **b, c** Bar, 25 µm.

Supplementary Movie legends

Supplementary Movie S1. Spontaneously beating ACMs. ACMs were co-cultured with ventricular myocytes for 20 days. See Fig. 1d for the phase-contrast image. Four ACMs are beating individually.

Supplementary Movie S2. Spontaneously beating ACMs constructing large assemblies. ACMs were co-cultured with ventricular myocytes for 35 days. See Fig. 1e for the phase-contrast image. Two cell assemblies are beating individually.
